# Optical Fiber Gratings Immunoassays

**DOI:** 10.3390/s19112595

**Published:** 2019-06-07

**Authors:** Médéric Loyez, Maxime Lobry, Ruddy Wattiez, Christophe Caucheteur

**Affiliations:** 1 Proteomics and Microbiology Department, Université de Mons, Champ de Mars 6, 7000 Mons, Belgium; mederic.loyez@umons.ac.be (M.L.); ruddy.wattiez@umons.ac.be (R.W.); 2 Electromagnetism and Telecom Department, Université de Mons, Bld Dolez 31, 7000 Mons, Belgium; maxime.lobry@umons.ac.be

**Keywords:** biosensing, tilted fiber bragg gratings, optical fibers, biomarker, immunoassays

## Abstract

Optical fibers are of growing interest for biosensing, especially for point-of-care and biomedical assays. Their intrinsic properties bestow them sought-after assets for the detection of low concentrations of analytes. Tilted fiber Bragg gratings (TFBGs) photo-inscribed in the core of telecommunication-grade optical fibers are known to be highly-sensitive refractometers. In this work, we present different strategies to use them for label-free immunoassays. Bare, gold-sputtered, gold-electroless-plated (ELP) and hybrid configurations are biofunctionalized with antibodies, aiming at the detection of cancer biomarkers. We discuss the relative performances of the tested configurations and show that each leads to singular key features, which therefore drives their selection as a function of the target application. The most sensitive configuration presents a limit of detection of 10^−12^ g/mL in laboratory settings and was successfully used ex vivo in freshly resected lung tissues.

## 1. Introduction

Fiber-optic sensors have known cutting-edge technological improvements in recent years [1]. Additionally to their flexibility, reliability, ease of use, and miniaturized size, multidisciplinary developments in both biochemical sciences and engineering have mainly contributed to novel and innovative outputs [2,3]. Many optical fiber devices have already proven their skills in biosensing, especially with the following architectures: unclad fibers [4,5], tapered fibers [6], long period fiber gratings (LPFGs) [7,8], fiber Bragg gratings (FBGs) [9,10], and also bend-etched/U-bent fibers, among others [11,12,13].

These architectures are usually associated to thin metal coatings to generate surface Plasmon waves and increase the sensitivity to surrounding refractive index (SRI) changes [14]. These collective plasmonic oscillations happen at a metal–dielectric interface when the component of the light along the interface and its polarization state match those of a plasmon wave [15]. 

When receptors such as antibodies are grafted on the metal film, biological sensing is possible in small volumes of analytes, at concentrations starting from 1 pM or even lower [16,17]. It is also possible to use these sensors in non-liquid media such as gel matrices or resected human tissues, expanding their applicability [18]. 

Many biochemical strategies can be implemented on the fiber surface in order to detect targets of different natures (DNA, proteins, cells, etc.) [19]. The use of antibodies is often presented as the gold standard to detect proteins in many assays while other approaches such as the synthesis of aptamers [20,21], nanobodies [22], molecular imprinted polymers [23] are gaining interest to increase stability, sensitivity, and/or specificity.

In our work, we make use of tilted fiber Bragg gratings (TFBGs) permanently photo-inscribed in the core of telecommunication-grade and single-mode optical fibers [24]. TFBGs are short-period gratings yielding a backward coupling of the light from the core to the cladding. TFBGs couple the core-guided light into tens of backward-going cladding modes, providing comb-like transmitted amplitude spectra featuring discrete and narrowband (full width at half maximum < 200 pm) resonances [25]. When P-polarized light illuminates the sensing interface, the surface plasmon resonance is revealed by a unique spectral feature that can be accurately tracked as a function of SRI changes. This has been reported both in liquids and gas [10,26]. It is therefore possible to use the intrinsic properties of TFBGs for biosensing in the outer medium directly into contact with the silica surface [27]. 

The thickness and homogeneity of the metal deposited on the fiber surface are the main parameters to control to obtain a clear surface plasmon resonance (SPR) excitation [28]. According to the literature and our previous works, it is known to happen for coating thicknesses between 30 and 70 nm. The most widespread techniques to metal-coat optical fiber surfaces for SPR excitation are evaporators or sputter-coaters. As depositions are made in a chamber under vacuum, the optical fiber signal cannot be used to monitor the evolution and the quality of the deposition. Usually, this is done extrinsically using an online quartz microbalance, which therefore may cause important differences between the measured thickness and the one really applied on the fiber surface. Oppositely, the electroless plating (ELP) allows an online-monitoring of the fiber signal and is therefore gaining interest for use with optical fibers [29]. The direct ELP of gold relies on the immersion of silanized optical fibers into a plating solution, where gold salts and reducing agents progressively form a gold film onto the target surface, without the need for external power contribution. [30] Finally, the use of gold nanoparticles as amplification-labels on the optical fiber surface is also known to enhance the sensitivity of the system, especially when they are used as sandwich-assays or when they are specifically bound to the fiber surface [31,32,33]. The structures and shapes of these gold particles or gold films such as those obtained with ELP can lead to hybrid phenomena, resulting in localized(-like) surface plasmon excitations (LSPR) instead of pure SPR obtained with continuous metal sheaths [34]. This phenomenon extends the sensitivity to “near-surface” interactions [35,36,37]. 

In this article, we overview four different TFBGs configurations deployed for immunosensing purposes: bare TFBGs (1), coated TFBGs with a sputtered gold layer (2), an electroless-plated gold layer (3), and a hybrid coating combining sputtered and electroless-plated gold layers (4). The bio-immobilization strategies are adapted and optimized according to the substrate, as described below. To obtain a fair comparison between their relative performances, they are oriented towards the detection of the same protein, namely cytokeratin 17 (CK17), which is a relevant biomarker selected for cancer diagnosis [38,39]. 

Assays were conducted in vitro with growing concentrations of CK17 starting from phosphate buffer saline supplemented with 10% of serum. Hence, we believe that our findings are relevant to orient the choice towards the best-suited configuration according to the foreseen application, both in terms of production process and obtained performances. The most sensitive architecture in terms of limit of detection was finally successfully validated ex vivo for the detection of CK17 in freshly biopsied lung tissue [40]. 

## 2. Materials and Methods

### 2.1. Materials

Phosphate Buffer Saline (PBS) came from Thermo Fisher Scientific (Waltham, MA, USA). Sodium borohydride (NaBH_4_), (3-aminopropyl) trimethoxysilane 97% (APTMS), N-hydroxysuccinimide (NHS), Gold nanoparticles 10 nm in citrate buffer (AuNPs), Gold(III) chloride trihydrate (HAuCl_4_·3H_2_O), Sulfate hydroxylaminum ((NH_2_OH)_2_·H_2_SO_4_) and N-(3-Dimethylaminopropyl)-N′-Ethylcarbodiimide Hydrochloride (EDC) were purchased from Sigma-Aldrich (St. Louis, MO, USA). Anti-Cytokeratin 17 antibodies, and Cytokeratin 17 (CK17) full human proteins came from Abcam (Cambridge, UK). Bovine Serum Albumine (BSA) was purchased from Acros Organics™ (Thermo Fisher Scientific, Waltham, MA, USA). All buffers were prepared freshly in deionized water and protein solutions were prepared in PBS at pH 7.2, and preserved at 4 °C. Telecommunication-grade optical fibers (SMF-28, single-mode silica OF Ø 250 µm with the polymer jacket, Corning Inc., Corning, NY, USA) were used to connect each sensor, made in photosensitive PS-1250 optical fiber. 

### 2.2. Tilted Fiber Bragg Gratings Manufacturing

TFBGs were manufactured using a NORIA Laser (NorthLab Photonics, Nacka, Sweden) in photosensitive single-mode optical fiber (PS-1250 from Fibercore, Southampton, UK). The grating manufacturing was performed with a deep ultraviolet pulsed excimer laser (Coherent Existar XS 500 Hz-ArF at 193 nm, Coherent Inc., Santa Clara, CA, USA) using the phase-mask technique to achieve ~7° tilted gratings. 

### 2.3. Optical Fiber Interrogation

**Figure 1:**
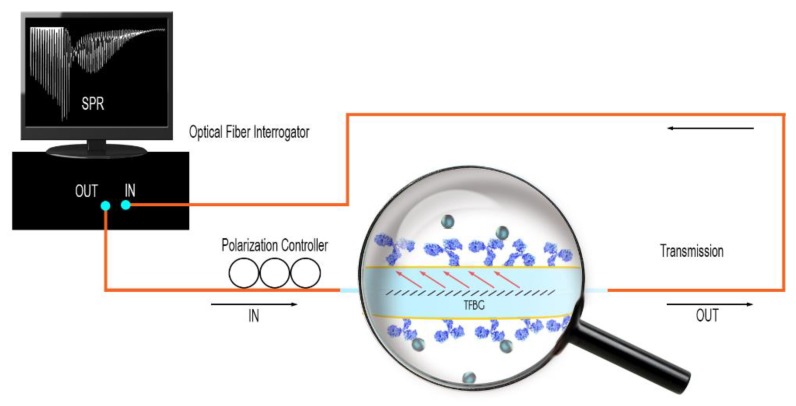
Scheme of the experimental setup in transmission. The gold-sputtered tilted fiber Bragg grating (TFBG) is connected using pigtails (SMF-28) to a polarization controller and an optical interrogator.

All the experimental data reported were obtained in transmission, using an FBG interrogator from FiberSensing (HBM, Darmstadt, Germany) with a scanning rate of 1 Hz and a wavelength resolution of 1 pm. The optical fibers were immobilized on a fixed horizontal support and immersed successively in different liquid samples (from blank to the highest protein concentration). A polarization controller was added between the sensor and the interrogator for the sputter-coated sensors (Figure 1), to allow the acquisition of the optimum state of near-IR light polarization (P-mode). When working with electroless plated gold surfaces, the interrogation was made using an optical vector analyzer to record the polarization dependent loss (PDL) spectrum, as explained in Section 3.1. PDL is defined as the ratio between the maximum and minimum optical powers recorded when the input state of polarization is varied over all possible states.

### 2.4. Description of the Four Different Sensing Configurations

#### 2.4.1. Bare-TFBGs

The TFBG surface was first cleaned by immersing the fiber into a piranha solution made of H_2_SO_4_/H_2_O_2_ (ratio 4:1) during 15 min. Then, a silanization process was carried out to form a silane film on the surface, using immersion in (3-aminopropyl)trimethoxysilane (APTMS) 1% in methanol for 20 min at room temperature.

**Figure 2:**
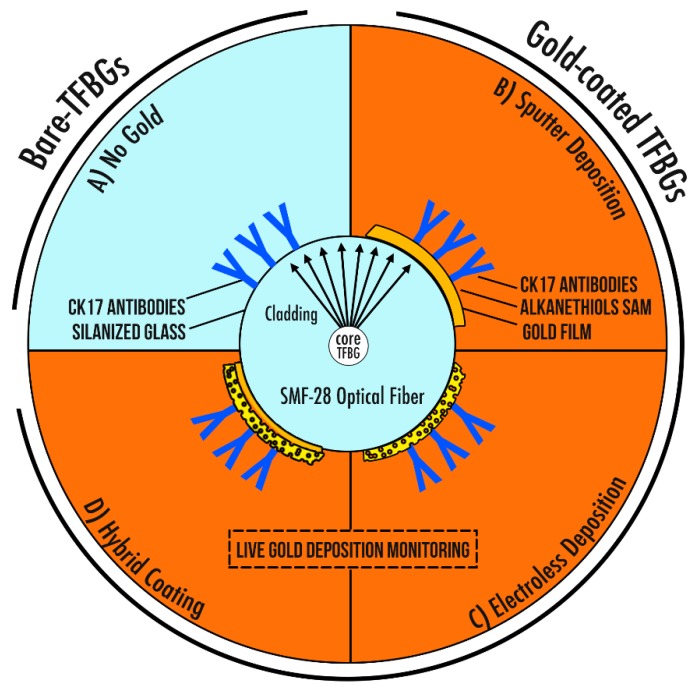
Scheme of the four studied sensing configurations. (**A**) The bare-TFBGs with adsorbed antibodies on the silica surface. (**B**) The sputter deposition under vacuum and the covalent bonding of anti-CK17 antibodies on the gold film. (**C**) The electroless deposition of gold and the covalent bonding of anti-CK17 antibodies on the agglomerated Au particles. (**D**) A hybrid-coating coupling a deposition of 4 nm Au by sputter-coater followed by an ELP deposition, which is functionalized using covalent bonding of anti-CK17 antibodies.

The silanized fibers were directly immersed into anti-CK17 (20 µg/mL in PBS) for 2 h at room temperature. After that, they were gently rinsed using PBS and dried under N_2_. This protocol was adapted from [41,42] (Figure 2A).

#### 2.4.2. Gold-Coated TFBGs: Sputter Deposition

First, a gold deposition of ~50 nm was performed by sputter-coater with argon under vacuum (10^−4^ mbar), and a thermal annealing at 200 °C was applied during 1 h 30 min to increase the adhesion of the gold film on the silica surface and enhance its efficiency for biosensing. [43] The gold layer was sputtered in two steps with a rotation of the samples between the two depositions. SPR-TFBGs were then gently cleaned in absolute ethanol and stored in dry conditions.

A self-assembled monolayer of dithiols was immobilized on the gold film (S_2_PEG_6_COOH 2 mM in ethanol, overnight at RT). Then, the optical fibers were immersed in NHS (N-hydroxysuccinimide 0.1 M)/EDC (N-(3-Dimethylaminopropyl)-N′-Ethylcarbodiimide Hydrochloride 0.5 M) in pure water for 20 min. After that, they were immersed into a CK17-antibodies solution (20 µg/mL in PBS buffer) during 1 h 30 min. (Figure 2B)

#### 2.4.3. Gold-Coated TFBGs: Electroless Deposition

The optical fibers were cleaned using a piranha solution H_2_SO_4_/H_2_O_2_ (ratio 4:1) for 10 min at room temperature. They were then rinsed in mQ water and immersed in APTMS 1% solution (in methanol) for 20 min. They were then rinsed with methanol and dried in the oven during 15 min at 80 °C. After that, the sensors were immersed in a commercial solution of 10 nm-diameter AuNPs for 1 h at room temperature without stirring.

They were then placed in the plating solution of 0.4 mM NH_2_OH·H_2_SO_4_ and 3 mM HAuCl_4_·3H_2_O. The plating was recorded using the spectrum analyzer (with PDL measurements). 

Once plated, fibers were biofunctionalized following the same procedure as for the sputtered optical fibers, involving covalent bonding of antibodies (Figure 2C).

#### 2.4.4. Hybrid Gold-Coated TFBGs: Mix of Sputtering and ELP

A first gold coating of ~4 nm (according to build-in quartz microbalance) is deposited by sputtering on both sides of TFBGs. 

These coated gratings are then directly immersed into the gold plating solution. The thin gold layer deposited by sputtering is sufficient to initiate the ELP reaction without the need for silanization or gold particles addition. The electroless plating is then performed as mentioned in paragraph 2.4.3. 

All these four sensing configurations were then blocked using BSA 5% in PBS at room temperature for 1 h 30 min.

### 2.5. Sensing Experiments

All the experiments were performed in laboratory settings. The biofunctionalized optical fibers were first kept straight with two clamps and brought into contact with PBS, used as a reference measurement. Once the polarization state and the acquisition settings were set, growing concentrations of CK17 proteins in PBS were brought on the TFBG surface using micropipettes (small volumes of 500 µL). The signal was monitored during 5 min in each solution and data were acquired through an acquisition program developed with the LabView software.

## 3. Results and Discussion

### 3.1. Spectral Sensitivity

**Figure 3:**
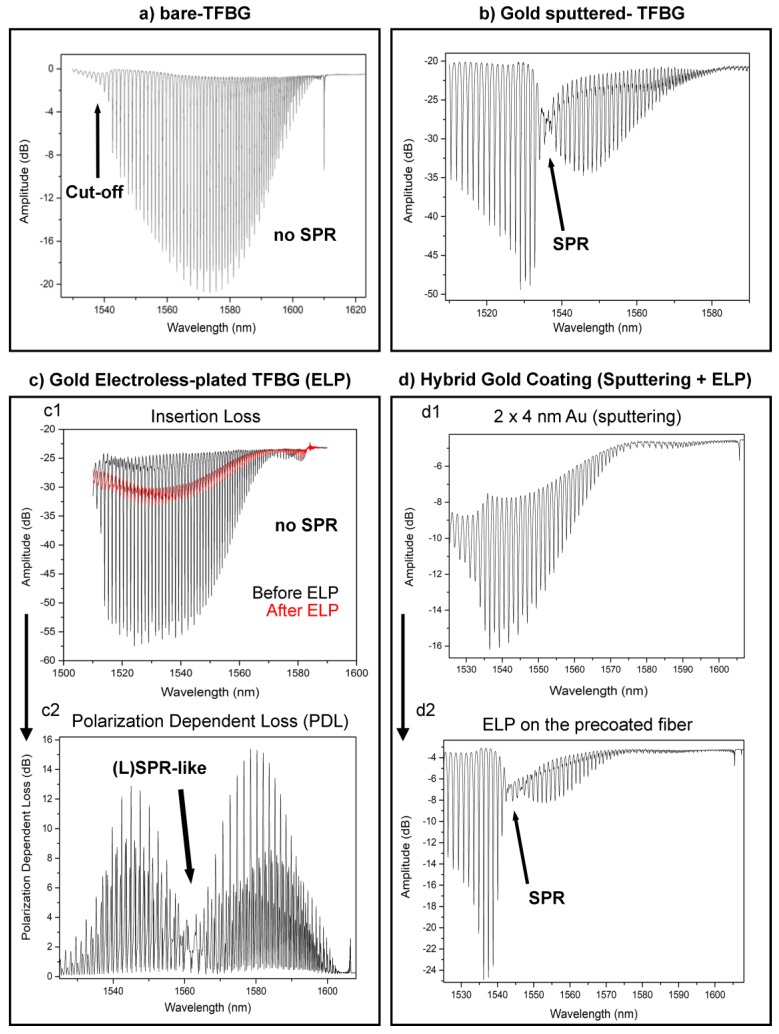
(**a**) Bare-TFBG amplitude spectrum monitored in PBS with the sensing part located at the cut-off area. (**b**) P-polarized spectrum of a sputter-coated TFBG with the most sensitive modes located at lower neighbor wavelengths of the SPR attenuation. (**c1**) Transmitted amplitude spectrum before and after ELP process. (**c2**) The same plating leads to a pronounced change in the PDL spectrum. (**d1**) The deposition of a ~4 nm-thick gold layer by sputtering does not lead to SPR attenuation. (**d2**) While the ELP is performed on that layer, the reaction can be stopped at the optimum point to reach SPR excitation using classical insertion loss interrogation.

When using bare TFBGs, the spectral acquisition can be made without taking care of the state of polarization as these gratings are weakly sensitive to polarization effects. The maximum sensitivity to SRI changes is obtained for the so-called cut-off mode, i.e., the cladding mode resonance for which the effective refractive index is just above the one of the surrounding medium. (Figure 3a) Tracking the wavelength shift of this mode reveals the intrinsic properties of TFBGs. 

The immobilization of a gold sputtered film on the surface of the TFBG allows the generation of SPR while the state of polarization is finely controlled, by the addition of a polarization controller inbetween the sensor and the light source. This configuration presents a singular feature consisting of cladding modes attenuation, revealing SPR excitation. The most sensitive part of the spectrum is located at this point and the neighbor modes are usually tracked for biosensing, as done in [16]. (Figure 3b) The relatively smooth surface obtained by this method allows the achievement of a well-defined attenuation yielding maximum sensitivity. 

The gold electroless deposition is a more flexible process. Many parameters such as the gold concentration, the rapidity of the deposition and the pre-treatment of the glass can be modified. Its most important asset is the possibility to track the deposition of the metal in real time and evaluate its effect on the TFBG spectral content. It is therefore easier to optimize and to refine the plating time to achieve the highest sensitivity. This being said, our developments on the ELP process have rapidly confirmed that a classical measurement of the amplitude spectrum was not the optimum read-out technique to ensure this refinement. Figure 3c1 compares the signals measured before and after 3 min of ELP. Only an overall attenuation of the cladding mode resonances can be noticed, probably resulting from a scattering of light by the growing nanoparticles. Additionally, adjusting the correct state of polarization online is really challenging. 

For that reason, PDL measurements were adopted. In the case of FBGs, it was shown that PDL corresponds to the absolute value of the difference between the two orthogonally-polarized amplitude spectra [44]. Therefore, for gold-coated TFBGs, the PDL spectrum perfectly reveals the difference between P-polarized (azimuthal modes that tunnel inside the gold layer and excite the SPR) and S-polarized (radial modes that are reflected by the gold layer) transmitted amplitude spectra. This yields a singular feature (Figure 3c2) when the plating time is sufficient. The local attenuation of the PDL spectrum is the most sensitive area to SRI changes and will therefore be used for further biosensing experiments.

The main benefit of the hybrid gold-coated TFBG configuration is the live monitoring of the TFBG signal, allowing to achieve an optimum gold thickness and a similar SPR attenuation as the one obtained for sputtered configurations. This is unique and makes the technique highly reproducible. A second asset is the decrease of the preparation time, as there is no need for silanization or gold nanoparticles immobilization. Also, classical polarization-assisted interrogation settings are used, as for sputter-coated TFBGs. (Figure 3d)

The resonances indicated by an arrow in Figure 3 are those that will be used in the following to demodulate the biosensor response.

### 3.2. Surface Influence on the Plasmonic Response

**Figure 4:**
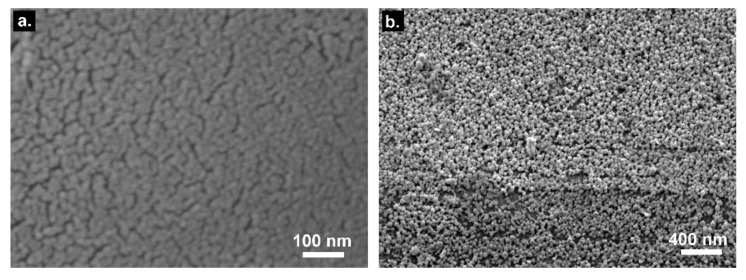
(**a**) SEM picture of a sputter-coated optical fiber surface (1 × 50 nm Au). (**b**) SEM picture of an electroless-plated (ELP) optical fiber surface.

A scanning electron microscopy (SEM) analysis shows the typical smooth structure achieved using the sputtering deposition. The layer is overall homogeneous in terms of thickness and surface coverage, which therefore yields a well-defined SPR signature (Figure 4a). On the other hand, the ELP process depicts a surface composed by agglomerations of growing gold particles with local areas of uncovered—or less covered—silica (Figure 4b). Hence, we can consider that the PDL signature is the spectral manifestation of a phenomenon close to a localized SPR rather than a pure SPR. It is worth mentioning that this gold layer is also strongly bound to the surface thanks to the silanization process, preventing the need for a thermal annealing process as required for sputtered surface to ensure sufficient metal adhesion during the successive immersions in liquid samples.

**Figure 5:**
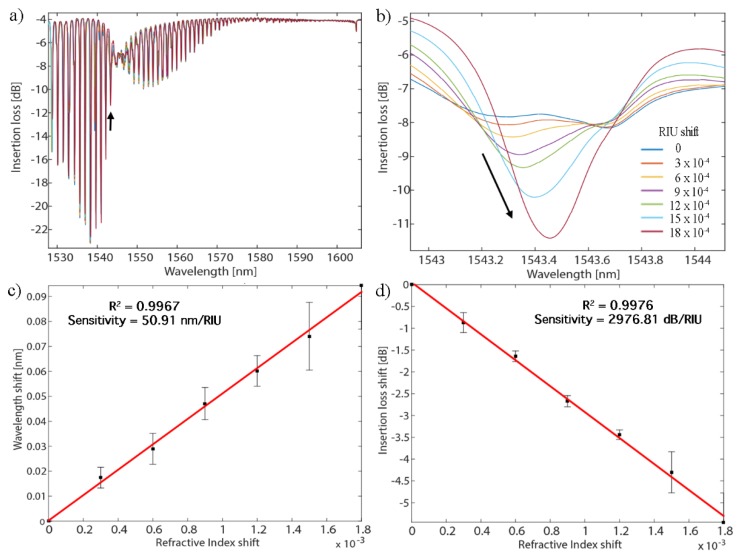
(**a**) P-polarized spectral evolution of a hybrid gold-coated TFBG configuration immersed in different LiCl solutions. (**b**) Zoom on the most sensitive mode. (**c**) Wavelength evolution of that mode as function of RI change (**d**) Amplitude evolution of that mode as function of refractive index (RI) change for three tested fibers.

The refractometric sensitivity of the hybrid configuration was computed using calibrated LiCl solutions. This yields a value of 50.9 nm/RIU for the wavelength shift of the most sensitive mode and 2976.8 dB/RIU for its amplitude change (Figure 5), which is similar to values reported for gold-sputtered TFBGs [10].

The following section will now focus on biofunctionalized configurations. Prior to present the biosensing results, we will present our experimental approach to analyze the immobilization efficiency. 

### 3.3. Analysis of the Biofunctionalized Surface

The amount of immobilized receptors on the optical fiber surface and their relative activity drive the biodetection performances. In order to evaluate the quality of the functionalization process, we rely on confocal microscopy using fluorescent-tagged antibodies (fluorescein isothiocyanate, FITCs) and a theoretical model based on molecular parameters.

**Figure 6:**
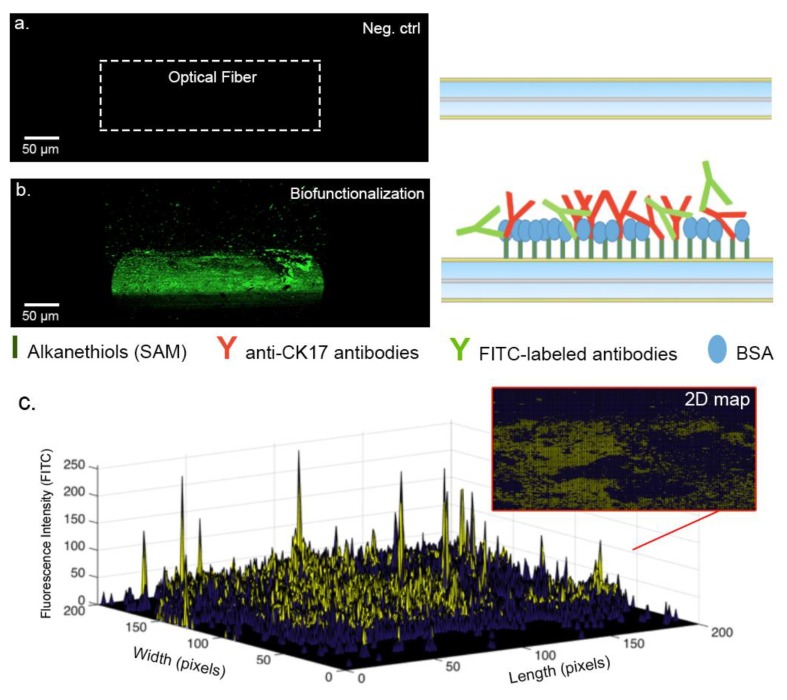
Confocal microscopy analysis performed using fluorescein isothiocyanate (FITC)-labeled antibodies. (**a**) Scans performed with a gold-coated SMF28 optical fiber (no fluorescent antibodies). (**b**) Scan performed with a gold-coated optical fiber covered with the FITC-labeled antibodies and Bovine Serum Albumine (BSA)-blocked. (**c**) 3D map obtained by the fluorescence intensity, bringing approximations of the antibodies covered areas.

The 3D confocal microscopy experiments reveal the presence of covalently-bonded FITCs antibodies that appear green (Figure 6a,b). Images were then analyzed to count the number of illuminated pixels and their relative intensity. (Figure 6c). This study revealed an estimated covered surface between 35% to 45% of the total grating surface, meaning that the experimental number of grafted receptors is close to 1.6 × 10^10^. This number overestimates the amount of well-oriented antibodies, which are considered to represent 4% to 10% of the total number of immobilized receptors [45,46].

To validate this experimental result, we can also estimate a theoretical value. To this aim, knowing that a concentration of 20 µg/mL of antibodies is used for the experiments, we consider that ~8 × 10^13^ antibodies per mL are available for the functionalization process. As the area of the cylindrical sensor is 4 mm^2^, the number of target proteins in the tested sample is ~3 × 10^12^ and the projected area of an antibody is 10 nm^2^, we can therefore assume that the optical fiber grating surface is theoretically covered with ~4 × 10^10^ interaction units. This theoretical value—corresponding to the best case—confirms that the value estimated by confocal microscopy is sound. 

These values have driven the test protocol used in Section 3.4, where growing concentrations of the target protein were used up to 10^−6^ g/mL, for which they are in excess compared to the reactional sites.

Contrary to expectations, it is important to mention that a too important surface coverage of the receptors can be more a constraint rather than a benefit, as the neighboring molecules can provoke steric hindrance and therefore negatively impact the interactions with the targets, even if receptors are well oriented [47,48].

### 3.4. Biodetection of CK17 Proteins

The experimental immunoassays were performed in laboratory settings using the four aforementioned optical fiber architectures. 

**Figure 7:**
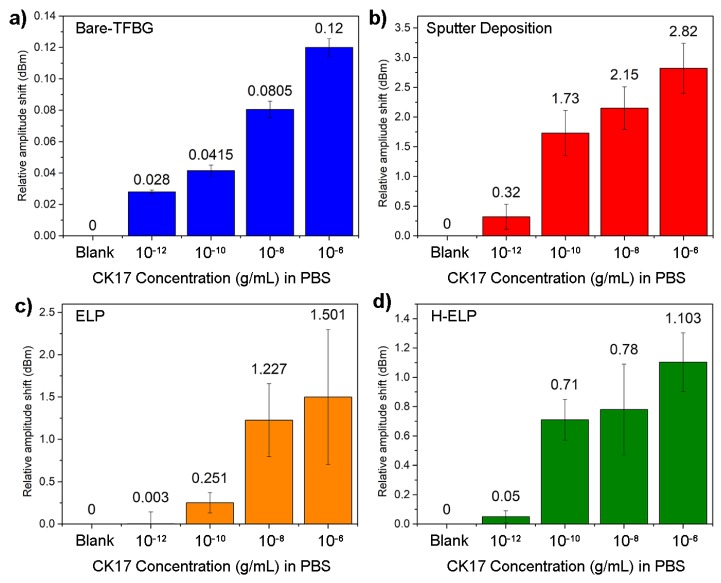
Cytokeratin-17 detection in Phosphate Buffer Saline (PBS). (**a**) Detection using the sensitivity of the cut-off area of bare-TFBGs. (**b**) Detection through the SPR-related modes of gold sputter-coated TFBGs. (**c**) Detection through the sensitive modes of the polarization dependent loss (PDL) spectra of gold-ELP-TFBGs. (**d**) Detection through the hybrid configuration. Data show means of amplitude shifts ± standard deviations for three tested fibers, in each condition.

Functionalized bare-TFBGs were successively immersed into growing CK17 concentrations in PBS, from 10^−12^ g/mL to 10^−6^ g/mL. Their transmitted amplitude spectrum features local amplitude changes at the cut-off region of the spectra. The relative amplitude shifts were computed after 5 min of immersion in each concentration, with reference to the pure PBS. The processed information is displayed in Figure 7a. Error bars correspond to the standard deviation computed for three experiments performed in the same experimental conditions.

Figure 7b displays the relative amplitude change of the most sensitive mode of the functionalized sputtered TFBG configuration. As expected, the changes are more significant, with an easy-to-detect amplitude variation for the smallest tested concentration at 10^−12^ g/mL. It is worth mentioning that the thermal annealing process also ensures sufficient gold film adhesion and therefore confirms its potential for use in complex environments or samples yielding higher mechanical stress.

Figure 7c focuses on the results obtained with ELP-TFBGs. Overall, these structures present an intermediate behavior between bare and gold-sputtered configurations. Error bars are more significant and the limit of detection is higher than for gold-sputtered TFBGs. It is computed to be 10^−9^ g/mL. Our observations confirm that the PDL interrogation is a relevant demodulation technique in terms of plasmonic biosensing for ELP TFBGs. The cost-effectiveness of the process, its online monitoring and the subsequent strong gold adhesion remain incontestable gains for this sensing architecture.

The hybrid-ELP process shows interesting detection limits with a similar behavior to sputter-coated TFBGs. The live monitoring of the gold deposition allows a fine control of the plasmonic state, which finally drives the quality of the biodetection performances. Significant shifts occur around 10^−10^ g/mL.

All these measurements were compensated for potential temperature fluctuations during experiments, by reference to the Bragg mode (corresponding to the core mode coupling) at the right end of all spectra that is not sensitive to surrounding refractive index changes.

### 3.5. Detection of CK17 Proteins in Resected Human Lung

In vitro experiments have confirmed that the most relevant configuration to perform immunoassays with the highest sensitivity and the best signal-to-noise ratio in complex conditions remains gold-sputtered TFBGs. As the Cytokeratin-17 protein was identified as potential cell-surface biomarker for lung cancer, we have conducted some experiments with the optrodes inserted in a protective packaging, directly into resected tumors [13].

**Figure 8:**
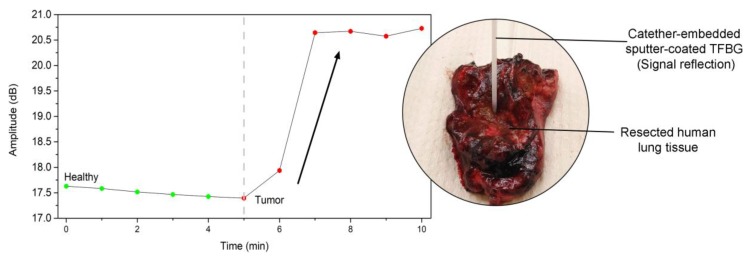
Graph showing the biosensor response in both healthy and tumorous parts of a resected human lung tissue. The biosensor was used at the tip of a catheter.

To allow its insertion into a catheter and to optimally exploit the sensitive area at the tip of the sensor, the optical fibers were cleaved after the TFBG and a silver mirror was added to enhance the signal reflection. Thanks to an effective surface blocking and after a thorough determination of the noise threshold, the sensors were able to screen the presence of CK17 in tissue (Figure 8).

The optical fibers were firstly inserted in the healthy part of the tissue and then in the tumorous part of the resected sample. The first insertion in the healthy part plays the role of the reference for the following tumorous monitoring.

## 4. Conclusions

Optical fiber gratings—especially when associated with metal coatings—are increasingly studied for biosensing, both for the detection of analytes though biomedical analyses and for environmental and food controls. When industrial applications and/or mass production are targeted, there remains room fore improvement for a correct substrate preparation. In this road towards the production of robust biosensors, we have studied four configurations combining TFBGs, gold coatings and biofunctionalization strategies. We have presented an overview of their characteristics from their manufacturing to their use as biosensors. The bare-TFBG strategy shows the lowest sensitivity due to the absence of SPR enhancement but depicts interesting assets, especially because it can be used straightforwardly without taking care of polarization effects. The gold sputtered deposition leads to the highest sensitivity for the detection of our targets (experimental LOD close to 1 pg/mL) and is adequate for complex matrices analyses, as it was implemented inside a catheter and tested in resected lung tissues. The gold electroless plating remains a rapid method to generate a gold interface and allows a live monitoring of the gold deposition process, which presents high adhesion on the silica surface. ELP-sensors are also able to significantly detect concentrations as low as 1 ng/mL of CK17, when the read-out is based on the PDL spectrum analysis. A fourth configuration, combining sputtering and electroless plating, depicts a sensitivity comparable to fully sputter-coated gratings while bringing relevant benefits in terms of production. All these optical fiber-based platforms can find their place in a wide variety of applications, driven by their own specifications.
